# Meeting an un-MET need: Targeting MET in non-small cell lung cancer

**DOI:** 10.3389/fonc.2022.1004198

**Published:** 2022-10-21

**Authors:** Elena Michaels, Christine M. Bestvina

**Affiliations:** ^1^ Department of Medicine, University of Chicago, Chicago, IL, United States; ^2^ Department of Medicine, University of Chicago Comprehensive Cancer Center, Chicago, IL, United States

**Keywords:** non-small cell lung cancer (NSCLC), MET exon 14, tyrosine kinase inhibitor, MET amplification, EGFR

## Abstract

The MET pathway can be activated by MET exon 14 skipping mutations, gene amplification, or overexpression. Mutations within this pathway carry a poor prognosis for patients with non-small cell lung cancer (NSCLC). MET exon 14 skipping mutations occur in 3-4% of patients with NSCLC, while MET amplifications are found in 1-6% of patients. The most effective method for detection of MET amplification is fluorescent *in situ* hybridization (FISH) and of MET exon 14 skipping mutations is RNA-based next generation sequencing (NGS). Immunohistochemistry (IHC) is an alternative method of diagnosis but is not as reliable. Early studies of MET tyrosine kinase inhibitors (TKIs) demonstrated limited clinical benefit. However, newer selective MET TKIs, such as capmatinib and tepotinib, have improved efficacy. Both drugs have an acceptable safety profile with the most common treatment-related adverse event being peripheral edema. One of the most frequent resistance mechanisms to EGFR inhibition with osimertinib is MET amplification. There is interest in combining EGFR inhibition plus MET inhibition in an attempt to target this resistance mechanism. Additional ways of targeting MET alterations are currently under investigation, including the bi-specific antibody amivantamab. Additional research is needed to further understand resistance mechanisms to MET inhibition. There is limited research into the efficacy of immune checkpoint inhibition for MET-altered NSCLC, though some data suggests decreased efficacy compared with wild-type patients and increased toxicity associated with the combination of immunotherapy and MET TKIs. Future directions for research will include combination clinical trials and understanding rational combinations for MET alterations.

## Introduction

In the last decade, targeted cancer therapy has become a pillar in the management of non-small cell lung cancer (NSCLC). Genomic testing allows clinicians to identify oncogenic drivers that guide treatment decisions (1). The National Comprehensive Cancer Network (NCCN) guidelines recommend testing for a specific set of biomarkers in patients diagnosed with advanced or metastatic NSCLC. Commonly tested biomarkers include EGFR, BRAF, ERBB2, and KRAS mutations; ALK, ROS1, and RET rearrangements; NTRK 1/2/3 gene fusions; PD-L1 expression; and MET exon 14 skipping mutations and amplification (2). Results from this testing are used to determine eligibility for novel therapies, which can improve both survival and quality of life for patients (3, 4).

The mesenchymal-epithelial transition (MET) oncogene is a receptor tyrosine kinase primarily expressed in epithelial cells (5). MET signaling is involved in the proliferation, invasion, and survival of cells. Gain-of-function MET alterations have been seen in several types of cancer, including NSCLC (5–7). These alterations occur as a result of point mutations, insertions, or deletions and promote cell survival and angiogenesis *via* induction of VEGF. MET alterations include exon 14 skipping mutations, gene amplification, and protein overexpression (7). Each of these alterations have been detected in NSCLC. They are associated with a poor prognosis (7). MET exon 14 skipping mutations are more common in elderly patients over the age of 70, women, and non-smokers (8). MET amplification is often diagnosed in patients under the age of 65 with a smoking history (9, 10).

MET directed therapies have emerged in recent years as treatment options for patients with advanced NSCLC with MET exon 14 skipping mutations and amplification. These treatments include both tyrosine kinase inhibitors (TKIs) and antibodies, and can target MET and the MET ligand, hepatocyte growth factor (HGF) (11). The GEOMETRY Mono-1 and VISION trials demonstrated improved clinical benefit for MET TKIs and led to the FDA approval of capmatinib and tepotinib, respectively. This review outlines the biology and detection of common MET alterations, summarizes currently available treatment options for patients with MET alterations, and identifies future directions for the use of MET TKIs in NSCLC.

## MET alterations

### MET exon 14 skipping mutations

MET exon 14 skipping mutations can occur through point mutations or genomic deletions that lead to a loss of exon 14. This increases protein stability by preventing ubiquitin-mediated degradation resulting in enhanced MET signaling and potential for malignancy (12, 13). The mutation occurs in 3-4% of patients with NSCLC (11).

### MET amplification

MET amplification is a result of focal gene amplification which causes an increase in gene copy number (GCN). Approximately 1-6% of patients diagnosed with NSCLC have a MET amplification. In addition to its role in certain malignancies, it has also been identified as a mechanism of acquired resistance for EGFR TKIs (14).

### MET alteration detection methods

Fluorescent *in-situ* hybridization (FISH) from solid tissue biopsy is the gold standard for detection of MET amplification (14, 15). The MET/CEP7 ratio can distinguish between true focal amplification versus polysomy of chromosome 7, which does not alter oncogenicity. Most studies use a cutoff MET/CEP7 ratio of ≥ 2 (13, 14).

Next generation sequencing (NGS) is an alternative to FISH that is becoming increasingly more common for diagnosis of MET amplification and exon 14 skipping mutations. A caveat to NGS is that there is no standardized method of detection and some assays do not control for CEP7. Studies have shown a discrepancy between diagnosis of MET amplification using FISH versus NGS (9, 14, 16). Generally, a cutoff with GCN ≥10 is preferred as it corresponds to a high level of MET amplification. The higher cutoff has been shown to have greater concordance with FISH. FISH is superior to NGS for detection of moderate to low levels of MET amplification (9, 16, 17).

Immunohistochemistry (IHC) has been studied and used to detect MET overexpression. However, IHC is not a reliable predictor of MET alterations such as exon 14 skip mutations or amplification (18). In a small cohort study of 181 patients, use of IHC to diagnose MET alterations was compared to FISH and NGS. A total of 3 out of 181 patients were diagnosed with MET amplification, 2 *via* FISH and 1 *via* NGS. Two out of three of these same patients screened negative for MET amplification based on IHC results. In addition, 71 of 181 patients screened positive for a potential MET alteration based on MET IHC but only 1/71 had a confirmed MET amplification and 2/71 had a MET exon 14 skipping mutation (18). Similar findings were shown in a study evaluating MET overexpression by IHC in diagnosing MET alterations in lung sarcomatoid carcinomas compared to FISH. There was a 50% sensitivity for IHC, 83% specificity, and a positive predictive value of 21.4% (19).

MET exon 14 skipping mutations are best diagnosed using DNA-based or RNA-based NGS (20). It is posited that RNA-based sequencing may be superior to DNA due to the ability to detect a wide array of mutational variants that may not affect or alter a splice site. One study comparing DNA versus RNA-based NGS for detection of MET exon 14 alteration found that 11 of 856 (1.3%) samples were positive using DNA-based sampling. RNA testing detected alterations in 17 of 404 (4.2%) patients. Furthermore, 286 samples were tested using both DNA and RNA-based sequencing. MET exon 14 alterations were detected in 10 samples *via* RNA testing. However, 6 of those samples were not detected using DNA (21). Additional studies have shown similar results demonstrating the superiority of an RNA-based approach to testing (22).

Liquid biopsy is an alternative diagnostic strategy to solid tumor biopsy that can identify MET alterations through NGS of circulating cell-free DNA (cfDNA) that is shed from solid tumors (23). In addition to diagnosing tumor-specific alterations to inform treatment decisions, liquid biopsy is also able to identify resistance mechanisms (15).

Regardless of the method used, detecting MET alterations can be difficult due to both the wide array of variants that can lead to altered MET expression and lack of a standardized approach to diagnosis.

## MET tyrosine kinase inhibitors

MET TKIs are sub-divided into three categories based on drug structure and the way in which the drug binds to MET. Type I MET TKIs are ATP-competitive inhibitors. Type I is further subdivided into type Ia and type Ib. Type Ia inhibitors bind to MET *via* the solvent front residue, G1163, and are known as non-selective MET TKIs as this residue is not specific to MET. Type Ib inhibitors function independent of this residue and selectively bind to MET alone. Due to their selectivity, type Ib MET TKIs have superior anti-tumor activity and a more tolerable safety profile. Type II MET TKIs are also ATP-competitive inhibitors but instead bind to the inactive form of MET. Type III MET TKIs bind allosterically outside of the ATP domain (24).

### Type 1a, non-selective MET TKIs

#### Crizotinib

Crizotinib not only exerts an inhibitory effect on MET, but also ALK, ROS, and RON. Crizotinib was originally approved for treatment of NSCLC with ALK or ROS1 rearrangements (25, 26). Additional studies of crizotinib have not been as promising for patients with MET alterations as they were for ALK or ROS1 rearrangements (27). The phase I trial, PROFILE 1001, first studied the role of crizotinib in treatment of advanced NSCLC patients with many genetic variants including MET alterations. Of the 69 patients enrolled with MET exon 14 skipping mutations, 65 were evaluable and there was an objective response rate (ORR) of 32% (95% CI 21-45) with median progression free survival (PFS) of 7.3 months (95% CI 5.4-9.1). Median duration of response (DOR) was 9.1 months (95% CI 6.4-12.7) (28). In addition, among 38 patients included in the study with MET amplification diagnosed by FISH, the high MET amplification group (MET to CEP7 ratio ≥ 4) had longer median PFS with crizotinib compared to patients with medium and low MET amplification (6.7 months vs 1.9 months vs 1.8 months) (29). Treatment-related adverse events (TRAEs) of any grade were common and seen in 94% of patients. The most common TRAE was peripheral edema in 51% of participants followed by GI symptoms and fatigue (28, 30). Twenty nine percent of patients experienced a TRAE of grade 3 or higher. The most common high-grade adverse events included elevated transaminases (4%) and dyspnea (4%) and one patient had treatment-related death due to interstitial lung disease (ILD) (28). PROFILE 1001 resulted in FDA breakthrough approval of crizotinib to treat MET alterations in NSCLC (**Table 1**).

**Table 1:** Published Trials of Type I MET TKIs.

Drug	Study, trial name	Population	Treatment	MET alteration	N	Objective response rate (ORR)	Progression free survival (PFS)
Crizotinib	NCT00585195, PROFILE-1001	Advanced NSCLC with MET exon 14 skipping mutation or MET amplification	Crizotinib 250mg BID	MET exon 14 skipping	65	32%	7.3 months
				Low amplification (MET/CEP7 ratio 1.8-2.2)	3	33.3%	1.8 months
				Medium amplification (MET/CEP7 ratio 2.2-4)	14	14.3%	1.9 months
				High amplification (MET/CEP7 ratio ≥4)	21	38.1%	6.7 months
	NCT02499614, METROS	Pretreated, advanced NSCLC with MET deregulation	Crizotinib 250mg BID	MET exon 14 skipping and MET amplification (MET/CEP7 ratio >2.2)	26	27%	4.4 months
	NCT02034981	Advanced NSCLC with c-MET ≥ 6 copies or c-MET mutations (including exons 14 and 16-19)	Crizotinib 250mg BID	c-MET ≥ 6 copies	25	16%	3.2 months
				c-MET-mutations	28	10.7%	2.2 months
			
Capmatinib	NCT02414139 GEOMETRY Mono-1	NSCLC with MET exon 14 skipping mutation or MET amplification	Capmatinib 400mg BID	MET exon 14 skipping, treatment naïve	28	68%	12.4 months
				MET exon 14 skipping, prior treatment	69	41%	5.4 months
				MET amplification, GCN ≥ 10, treatment naïve	69	29%	4.1 months
				MET amplification, GCN ≥10, prior treatment	15	40%	4.2 months
				MET amplification, GCN 6-9, prior treatment	42	12%	2.7 months
				MET amplification, GCN 4-5, prior treatment	54	9%	2.7 months
				MET amplification, GCN <4, prior treatment	30	7%	3.6 months
Tepotinib	NCT02864992 VISION	NSCLC with MET exon 14 skipping mutation or MET amplification	Tepotinib 500mg qd	MET exon 14 skipping MET amplification (GCN ≥2.5)	99 24	46% 41.7%	8.5 months 4.2 months
Savolitinib	NCT02897479	Unresectable or metastatic pulmonary sarcomatoid carcinoma or NSCLC with MET exon 14 skipping mutation	Savolitinib 600mg if ≥ 50kg or 400mg if <50kg	MET exon 14 skipping	61	49.2%	6.9 months

The METROS study, a phase II trial, investigated the efficacy of crizotinib in patients with MET dysregulation including MET exon 14 skipping mutations and amplification. In the MET cohort, ORR was 27% (95% CI 11-47) with median PFS of only 4.4 months (95% CI 3.0-5.8) (31). Similar results were seen for NSCLC patients with MET alterations enrolled in the phase II AcSé trial (32). Several ongoing phase II trials including MATCH and MATRIX continue to study the potential role of crizotinib in treating MET-altered NSCLC (**Table 2**).

**Table 2:** Active Trials of Type I MET TKIs.

Drug	Trial	Phase	Population	Treatment arms
Crizotinib	NCT02465060, MATCH	II	Advanced refractory NSCLC with MET amplification and MET exon 14 skipping mutation	Crizotinib PO BID
	NCT02664935, MATRIX II	II	NSCLC with ROS1 fusion, MET amplification, or MET exon 14 skipping mutation	Crizotinib PO BID
Capmatinib	NCT04427072, GeoMETry-III	III	Previously treated NSCLC with MET exon 14 skipping mutation	Capmatinib PO BID Versus Docetaxel IV q21d
	NCT04677595, GeoMETry-C	II	Advanced NSCLC with MET exon 14 skipping mutation	Capmatinib PO BID
	NCT04926831, Geometry-N	II	NSCLC with MET exon 14 skipping mutation or high MET amplification	Capmatinib PO BID
	NCT03693339	II	NSCLC with MET exon 14 skipping mutation	Capmatinib PO BID
	NCT02276027	II	NSCLC with c-MET gene alteration	Capmatinib PO BID
	NCT02323126	II	c-MET positive NSCLC	Capmatinib PO BID and nivolumab q2w
	NCT02750215	II	Advanced NSCLC with MET exon 14 skipping mutation who received prior MET inhibitor therapy	Capmatinib PO BID
Tepotinib	NCT04739358	I/II	MET-driven NSCLC with at least 1 intracranial lesion	Tepotinib PO qd alone Versus Tepotinib qd and concurrent TKI of choice
Savolitinib	NCT02117167, SAFIR02_Lung	II	MET-altered NSCLC	Savolitinib PO qd Versus Pemetrexed IV q3w
	NCT04923945	III	Locally advanced or metastatic NSCLC with MET exon 14 skipping mutations	Savolitinib PO qd

### Type 1b, selective MET TKIs

#### Capmatinib

Capmatinib was FDA-approved for treatment of NSCLC with MET exon 14 skipping mutations in 2020. This was based on data from the GEOMETRY Mono-1 study, a phase II clinical trial. The study enrolled 364 patients with confirmed MET exon 14 skipping mutations or MET amplification. Patients were further stratified based on prior treatment history. Among patients with MET exon 14 skipping mutations, 69 received prior therapy and had an ORR of 41% (95% CI 29-53) and median PFS of 5.4 months (95% CI 4.2-7.0). In contrast, 28 treatment-naïve patients had an ORR of 68% (95% CI 48-84) and median PFS of 12.4 months (95% CI 8.2-NE). The time to respond to capmatinib was rapid for both groups, as short as first tumor evaluation at 6 weeks. Results suggested an increased benefit in the treatment naïve population (13).

Patients with MET-amplified NSCLC had limited response to capmatinib. The trial closed early for futility in patients with MET amplification with GCN <10. While tumor response was seen for patients with GCN ≥ 10, this still did not meet the threshold for clinical relevance (ORR 29%, 95% CI 19-41, median PFS 4.1 months, 95% CI 2.9-4.8 in 69 previously treated patients; ORR 40%, 95% CI 16-68, median PFS 4.2 months, 95% CI 1.4-6.9 in 15 treatment-naïve patients) (13).

Capmatinib had an acceptable safety profile. TRAEs occurred in 88% of patients who received treatment, with peripheral edema seen in 50% of patients. Grade 3 or higher adverse events occurred in 67% of participants, and again peripheral edema was the most common high-grade event. Serious TRAEs were seen in 13% of participants and 11% discontinued treatment as a result (13, 30, 33).

#### Tepotinib

The phase II VISION trial led to FDA approval of tepotinib in 2021 as a second-line therapy for patients with MET exon skipping NSCLC. A total of 152 patients with MET exon 14 skipping mutations were enrolled in the study and received treatment. There was an ORR of 46% (95% CI 36-57) based on independent review, and median PFS was 8.5 months (95% CI 6.7-11). There was no significant difference in response for patients who had received prior lines of treatment from those who had not (34). Response time was considered rapid, and the majority of patients had a response within 6 weeks of treatment initiation.

TRAEs occurred in 89% of patients. Peripheral edema was the most common adverse effect, occurred in 63% of patients, lead to dose reduction in 16% of patients, dose interruption in 18%, and discontinuation in 5%. Twenty-eight percent of participants experienced a grade 3 or higher adverse event. The most common high-grade event (7%) was peripheral edema, followed by increased amylase and lipase. Serious TRAEs were reported in 15% of patients and 11% discontinued treatment (30, 34, 35).

A sub-group analysis of 23 patients with MET exon 14 skipping mutations and brain metastases demonstrated a robust treatment response with an ORR of 47.8% (95% CI 26.8-69.4) and median DOR of 9.5 months (95% CI 5.5-NE). There were 15 patients evaluated using Response Assessment in Neuro-Oncology Brain Metastases (RANO-BM) criteria, 12 received prior chemotherapy. 7 patients had measurable disease per RANO-BM and 8 had non-enhancing, non-target lesions. 9 had a partial response (PR), 3 with stable disease (SD), and 3 with progressive disease (PD) (36).

The efficacy and safety of tepotinib in an elderly population over the age of 75 with MET exon 14-altered NSCLC was further investigated in an additional group of patients and was consistent with the findings from the VISION trial (ORR 39.7%, 95% CI 28-52.3; median PFS 8.6 months, 95% CI 6.9-12.4) (35). Peripheral edema was the most common adverse event and occurred in 51.4% of the elderly patient population. Thirty four percent of patients over 75 had grade 3 or higher TRAEs and 14.7% discontinued treatment (35).

Results from the VISION trial in patients with MET-amplified NSCLC were analyzed separately. MET amplification was defined as GCN ≥2.5. A total of 24 patients were enrolled in the study with an ORR of 41.7% (95% CI 22.1-63.4) and median PFS of 4.2 months (95% CI 1.4-NE). Sixteen patients reported TRAEs of any grade and 7 of those patients were grade 3 or higher. Peripheral edema was again the most common adverse event and was reported in 37.5% of patients (37) (**Table 1**).

#### Savolitinib

Savolitinib was approved in China for conditional use in NSCLC with MET exon 14 skipping mutations following a multi-center phase II trial. The study enrolled 70 patients with confirmed MET exon 14 skipping mutations; 36% had pulmonary sarcomatoid carcinoma. There was an ORR of 49.2% (95% CI 31.1-55.3) in the tumor response evaluable set with a median PFS of 6.9 months and median OS of 14 months (38). Forty-six percent of patients experienced a high-grade TRAE and 24% were serious. Elevated liver enzymes and peripheral edema were the most common grade 3 or higher adverse events, and one patient died of tumor lysis syndrome which was attributed to savolitinib (38).

### Type II MET TKIs

#### Cabozantinib

Cabozantinib is another non-selective MET TKI that targets VEGFR1-3, RET, TIE2, FLT-3, KIT, and MET. It is currently FDA-approved for treatment of medullary thyroid cancer, renal cell carcinoma, and hepatocellular carcinoma. In a phase II trial, patients with advanced NSCLC with wild-type EGFR were randomized to receive cabozantinib alone, erlotinib alone, or erlotinib with cabozantinib. The study showed improved PFS in the cabozantinib alone group (4.3 months, HR 0.39, 80% CI 0.27-0.55) and the cabozantinib and erlotinib combination cohort (4.7 months, HR 0.37, 80% CI 0.25-0.53) compared to erlotinib alone (1.8 months, 95% CI 1.7-2.2) (39).

While the study had promising results, the trial did not test for MET alterations and it is unclear what role cabozantinib can play in the treatment of patients with MET alterations. One small study of patients with stage IV lung adenocarcinoma with MET exon 14 skipping mutations randomly assigned 8 patients to receive either crizotinib or cabozantinib. In the study only one patient ultimately received cabozantinib and had a complete response (CR) (40). More data is required to evaluate the efficacy of cabozantinib in MET-altered NSCLC. Results are awaited in the ongoing phase II trial, CABinMET (**Supplemental Table 2**) (41).

For information regarding additional Type II MET TKIs including merestinib, foretinib, and glesatinib and the Type III MET TKI, tivantinib, please see **Supplemental Tables 1**, **2**.

## MET antibodies

In addition to MET TKIs, there are several antibodies targeting MET that have been studied or are in development for treatment of NSCLC. Amivantamab is a bispecific antibody targeting EGFR and MET. It was first presented in the phase I CRYSALIS study, which included a population of NSCLC patients with EGFR exon 20 insertion. There was an ORR of 40% (95% CI 29-51) and median PFS of 8.3 months (95% CI 6.5-10.9). The most common adverse events were rash and infusion reactions, and the most common severe adverse event was hypokalemia (5%) (42). Updated results from the CHRYSALIS study were recently presented at the 2022 ASCO meeting including preliminary data from 55 patients with MET exon 14 skipping mutations. Among 22 treatment-naïve patients there was an ORR of 50%. An ORR of 17% was seen in patients with prior treatment. To date, 11 of the 15 patients who responded to amivantamab remain on treatment. These results suggest that amivantamab has anti-tumor activity for both EGFR and MET-altered NSCLC (43).

Early data from the phase II CHRYSALIS-2 trial was also presented at the 2022 ASCO meeting in which amivantamab was given in combination with the EGFR TKI, lazertinib, for patients with NSCLC who progressed on platinum-based chemotherapy and osimertinib. There were 162 patients who received treatment with an ORR of 33% and clinical benefit rate of 57% with median DOR of 9.6 months (44). The ongoing phase III MARIPOSA and MARIPOSA-2 trials are investigating amivantamab and lazertinib as potential first line therapy in EGFR-mutant NSCLC.

Emibetuzumab is a humanized IgG4 monoclonal bivalent MET antibody designed to block MET signaling. It was studied in a phase II trial in combination with erlotinib for treatment of stage IV, EGFR-mutated NSCLC. The study showed no significant difference in PFS for patients treated with the combination of erlotinib and emibetuzumab compared to erlotinib alone. However, *post hoc* analysis revealed that for 24 patients with markedly high MET expression there was a significant improvement in median PFS of 15.3 months in the combination group (45).

Onartuzumab is a recombinant humanized monoclonal monovalent antibody against MET. There have been several phase II and III clinical trials investigating the benefit of onartuzumab in combination with erlotinib and common chemotherapy regimens with no significant benefit in PFS or OS gained (46). Of note, while participants in these trials were tested for MET overexpression in their tumors, they were not tested for specific MET alterations such as MET exon 14 skipping mutations or amplification. Thus, it is possible that onartuzumab could have significant antitumor activity for patients with certain MET alterations, although this has not yet been studied.

More recently the antibody-drug conjugate (ADC), telisotuzumab vedotin, was developed. The ADC consists of a c-MET antibody linked to a microtubule inhibitor and was given FDA breakthrough therapy designation for EGFR wild-type NSCLC following initial data from the ongoing phase II LUMINOSITY trial. Tumors were tested for c-MET overexpression using IHC and subdivided into intermediate or high c-MET expression groups. Of the 136 patients who received treatment so far, there was an ORR of 52.2% in the c-MET high, EGFR wild-type group and 24.1% in the c-MET intermediate, EGFR wild-type group. Peripheral neuropathy, nausea, and low albumin levels were the most common TRAEs (47).

One other MET antibody under investigation is Sym015, which is made up of a mixture of 2 humanized antibodies (48).The antibodies bind non-overlapping epitopes on the SEMA-a domain of MET which promotes MET receptor internalization and degradation by preventing HGF from binding to MET (48). There is preliminary data on a phase II trial of Sym015 for treatment of NSCLC with MET amplification or exon 14 skipping mutation (48). Twenty patients were included in the expansion cohort with an ORR of 25% and disease control rate (DCR) of 80%. There was a response benefit for MET TKI naïve participants with an ORR of 50% and 100% DCR. Median PFS was 6.5 months for MET TKI naïve patients and 5.4 months for patients who received prior MET TKI therapy. The drug was considered safe with TRAE in 42.2% of patients, 13.3% of which were grade 3 or higher. The most common adverse events were fatigue and peripheral edema (48).

## Immunotherapy for patients with MET alterations

There is limited clinical benefit for use of immune checkpoint inhibitors (ICI) in patients with MET-altered NSCLC. Several studies have demonstrated modest efficacy (49, 50). This includes a multicenter, retrospective study in which 36 patients with MET alterations had an ORR of 16% to ICI therapy and median PFS of 3.4 months (95% CI 1.7-6.2) (50). Similar results were found in another study of 22 patients with MET exon 14 skipping NSCLC treated with ICI therapy (ORR 17%, 95% CI 6-36, median PFS 1.9 months, 95% CI 1.7-2.7) (49). There is no apparent correlation between biomarkers such as PD-L1 expression (>50%) or tumor mutational burden in predicting response to immunotherapy in this group of patients (49, 51).

Interestingly, one retrospective, multicenter study demonstrated an improved response to ICI therapy. Thirty patients with a MET mutation were evaluated and received either pembrolizumab or nivolumab. There was an ORR of 35.7% and median PFS of 4.9 months (95% CI 2.3-NE) (52). NCCN guidelines currently recommend single-agent targeted therapy for initial management of MET exon skipping NSCLC rather than chemotherapy or immunotherapy (2).

Genetic heterogeneity within MET-dysregulated NSCLC- including MET skipping mutation versus amplification, as well as co-occurring mutations, may affect response to immunotherapy. One study analyzed patients with NSCLC with MET exon 14 skipping mutations, MET amplification GCN≥10, and MET amplification GCN <10 for additional co-occurring mutations (53). Investigators found that there were more co-occurring mutations in MET-amplified tumors compared to the MET exon 14-mutated tumors, and that the type of co-mutation was dependent on the degree of MET amplification. The most common mutations were TP53, KRAS, and KEAP1. KRAS mutations were more common in MET-amplified tumors with GCN <10 while KEAP1 mutations were more frequent with GCN ≥10 (53). Patients with MET amplification with GCN ≥10 had worse median OS compared to GCN <10 (4 months vs 12 months, *p*=0.001) (53). While MET-amplified tumors with GCN ≥10 showed the worst OS of any cohort, treatment with immunotherapy after progression on first-line chemotherapy greatly improved OS compared to chemotherapy (36.0 months vs 4.0 months, *p*=0.004). A similar improvement in OS was seen with GCN <10 treated with ICI versus chemotherapy (19 months vs 8 months, *p*<0.0001). In contrast, OS was not statistically improved for patients with MET exon 14 skipping mutations (16 months ICI vs 10 months chemotherapy, *p*=0.147) (53). These findings suggest a difference in response to immunotherapy-based MET alteration subtype.

One reason immunotherapy may have limited efficacy in MET-altered NSCLC is through inhibition of stimulator of interferon gene (STING) signaling. The STING pathway promotes interferon (IFN) response and is integral in the recruitment of T-cells and NK-cells (54). A retrospective cohort study analyzed MET copy number and STING levels in patients previously diagnosed with NSCLC. Among 81 patients treated with anti-PD1 therapy following progression on first-line chemotherapy, those with the worst response to treatment were found to have high MET copy numbers and low IFNB. This suggested that MET amplification leads to impaired tumor immunogenicity and, therefore, reduces response to ICI (54).

The combination of a MET TKI with an ICI could potentially overcome resistance to immunotherapy. However, there is concern for increased toxicity with combination therapy. One phase II trial of NSCLC with high tumor PD-L1 expression (≥50%) randomized treatment-naïve patients 2:1 to receive combination pembrolizumab and capmatinib or pembrolizumab alone. The trial was terminated early due to toxicity concerns with combination therapy. At data cutoff, 51 patients were enrolled in the combination arm and 25 in the pembrolizumab-alone arm. Nineteen out of fifty-one patients (37.3%) in the combination arm discontinued treatment with 4 suspected deaths. Seven patients (28%) discontinued treatment in the single therapy arm (55). Further research is needed to determine the role of ICI therapy for treatment of patients with MET alterations both alone and in combination with targeted drugs.

## Acquired MET alterations in EGFR NSCLC

EGFR TKIs have been highly successful in the treatment of EGFR-mutant NSCLC. However, development of acquired resistance is common, and limits the long-term efficacy of this class of drugs. Approximately 60% of resistance to first generation EGFR TKIs is due to the T790M mutation which inhibits binding of TKIs to the ATP binding site of EGFR. Third generation EGFR TKIs such as osimertinib were subsequently developed to overcome common EGFR TKI resistance mechanisms such as the T790M mutation. MET amplification is another important mechanism of acquired resistance to EGFR TKIs that activates oncogenic signaling cascades downstream of EGFR through a bypass pathway (56–59). It occurs in approximately 30% of patients with progressive disease on EGFR TKIs (51). Importantly, MET amplification is one of the few known resistance mechanisms for third generation EGFR TKIs (58, 59).

Several trials have attempted to overcome resistance through combination therapy with MET TKIs. The INSIGHT study is a phase Ib/II trial that investigated the combination of tepotinib and gefitinib in patients with EGFR-mutant, T790M negative NSCLC with acquired resistance to EGFR TKI therapy compared to chemotherapy. While survival outcomes were similar overall for tepotinib and gefitinib compared to chemotherapy, sub-group analysis demonstrated a significant improvement in PFS and OS for patients with MET amplification treated with combination therapy (ORR 67% vs 43% median PFS 16.6 months vs 4.2 months, HR 0.13, 90% CI 0.04-0.43; median OS 37.3 months vs 13.1 months, HR 0.08, 90% CI 0.01-0.51) (60). A notably smaller survival benefit was shown for patients with high MET overexpression by IHC (ORR 68% vs 33%, median PFS 8.3 months vs 4.4 months, HR 0.35, 90% CI 0.17-0.74, OS 37.3 months vs 17.9 months, HR 0.33, 90 CI 0.14-0.76). This again suggests that the use of IHC to classify MET-altered NSCLC may inadequately identify the intended patient population which, in turn, impacts response to treatment. While overlapping toxicities with combination therapy is of concern, treatment was well tolerated in INSIGHT and the most common grade 3 or higher AEs were increased amylase (16%) and lipase (13%) (60).

Additional studies that utilized combination MET and EGFR TKIs to overcome MET-amplified EGFR TKI resistance include a phase Ib/II trial evaluating capmatinib and gefitinib and the phase Ib TATTON trial which included osimertinib in combination with savolitinib. Both showed similarly promising results to INSIGHT (**Table 3**) (61, 62). Continued studies of combination therapy to overcome MET-driven EGFR TKI resistance include the phase II SAVANNAH and ORCHARD trials of osimertinib and savolitinib in addition to INSIGHT2 with osimertinib and tepotinib (NCT03778229, NCT03944772, NCT03940703).

**Table 3:** Trials of MET TKIs in Acquired EGFR Resistance.

Study, trial name	Population	Treatment	MET alteration	N	Objective response rate (ORR)	Progression free survival (PFS)
NCT01982955, INSIGHT	EGFR-mutated NSCLC with MET overexpression or amplification with disease progression on EGFR TKI	Tepotinib 300mg or 500mg qd and Gefitinib 250mg qd Versus Standard chemotherapy	MET amplification	19	67% vs 43%	16.6 months vs 4.2 months
			MET overexpression	34	68% vs 33%	8.3 months vs 4.4 months
NCT02143466, TATTON	Advanced EGFR-mutated, MET-amplified NSCLC with disease progression on EGFR TKI	Savolitinib 300 or 600mg qd and Osimertinib 80mg qd	MET amplification	138	48%	7.6 months
NCT01610336	EGFR-mutated, MET-dysregulated NSCLC with disease progression on EGFR TKI	Capmatinib 400mg BID and Gefitinib 250mg qd	Total	100	29%	5.5 months
			MET amplification, GCN ≥6	36	47%	5.49 months
			MET overexpression	78	32%	5.45 months
NCT03778229, SAVANNAH	EGFR-mutated, MET+ NSCLC with progression on osimertinib	Savolitinib 300 or 600mg qd and Osimertinib 80mg qd	MET amplified or overexpressed	Active trial	Active trial	Active trial
NCT03944772, ORCHARD	EGFR-mutated, advanced NSCLC with progression on osimertinib	Savolitinib 300mg or 600mg qd and Osimertinib 80mg qd	None	Active trial	Active trial	Active trial
NCT03940703, INSIGHT 2	EGFR-mutated, MET-amplified NSCLC with acquired resistance to osimertinib	Tepotinib 500mg qd and Osimertinib 80mg qd Versus Tepotinib 500mg qd alone	MET amplification	Active trial	Active trial	Active trial

## Future directions

While MET TKIs have demonstrated clinical benefit and tolerable toxicity, the ability to diagnose and sequence MET alterations remains a challenge and there are currently no standardized methods to confirm MET alterations. In addition, as with all TKIs, duration of response can be limited by resistance. There are several proposed mechanisms of on-target and off-target resistance to MET TKIs. On-target mutations can affect drug-receptor binding and ATP inhibition (63, 64). Several mutations that confer resistance to type I MET TKIs include G1163R, which is associated with resistance only to crizotinib, D1228, and Y1230. Bypass pathways can also lead to off-target resistance *via* activation of oncogenic signaling cascades downstream from MET such as the MAPK and PI3K/AKT pathways. This can be mediated by mutations and amplifications in EGFR, KRAS, HER3, and BRAF (63, 64). Switching generations of MET TKIs may be feasible to overcome on-target resistance. One case report demonstrated the ability of cabozantinib to overcome a D1228 resistance mutation which was acquired during treatment with crizotinib. The patient presented had an initial PR at 6 weeks to cabozantinib but ultimately had PD after 4 months. Post-progression biopsy following treatment with cabozantinib did not detect the D1228 mutation (65). While these results are encouraging, the data thus far for type II and type III MET TKIs is not as promising as the selective MET inhibitors. Further understanding of resistance mechanisms and the role of combination therapy is needed.

There is an emerging role for combination therapy with MET and EGFR TKIs in EGFR-mutant NSCLC. Further research is needed to understand additional combination therapies in MET-altered NSCLC, including MET TKIs and ICIs. Several new agents targeting MET alterations are currently under investigation including the bi-specific antibody amivantamab. While not yet studied, such agents may be combined or used in place of MET TKIs to delay or prevent resistance.

## Conclusion

Treatment of NSCLC has significantly changed over the last decade with the rise of genetic testing and targeted cancer therapy. Activation of the MET pathway is an important oncogenic driver for many patients with NSCLC and has proven to be an effective target for therapy. The development of MET TKIs and, in particular, the selective MET TKIs tepotinib, capmatinib, and savolitinib, has altered the landscape of cancer treatment for an older population of patients who previously had limited treatment options outside of chemotherapy. The more recent emergence of MET antibodies including the bispecific antibody, amivantamab, is expanding upon available treatment options and is currently being studied as potential first-line therapy for EGFR-mutant NSCLC.

## Author contributions

All authors listed have made a substantial, direct, and intellectual contribution to the work and approved it for publication.

## Conflict of interest

CB reports the following disclosures: Advisory Board: AstraZeneca, BMS, Genentech, Jazz, JNJ, Novartis, Pfizer, Seattle Genetics, Takeda; Consulting Fee: Axiom Healthcare, Cardinal Health, Curio Science, CVS, OncLive. Targeted Oncology; Contracted Research: BMS; Speakers Bureau: Merck.

The remaining author declares that the research was conducted in the absence of any commercial or financial relationships that could be construed as a potential conflict of interest.

## Publisher’s note

All claims expressed in this article are solely those of the authors and do not necessarily represent those of their affiliated organizations, or those of the publisher, the editors and the reviewers. Any product that may be evaluated in this article, or claim that may be made by its manufacturer, is not guaranteed or endorsed by the publisher.
