# Supplementary material for: Meeting an un-MET need: Targeting MET in non-small cell lung cancer
Source: Front Oncol. 2022 Oct 21;12:1004198. doi: 10.3389/fonc.2022.1004198 (PMC9634070; doi:10.3389/fonc.2022.1004198)
Supplement: Supplementary file 1 [file DataSheet_1.docx]

Supplemental Table 1. Published Trials of Type II and Type III MET TKIs

| Drug | Study, trial name | Population | Treatment | N | Objective response rate (ORR) | Progression free survival (PFS) |
| --- | --- | --- | --- | --- | --- | --- |
| Cabozantinib | NCT01708954, ECOG-ACRIN1512  NCT01866410  NCT00940225  NCT00596648 | Advanced, pretreated, EGFR wild-type NSCLC  Advanced, EGFR-mutant NSCLC with progression on EGFR TKI  Pretreated NSCLC  EGFR-mutant NSCLC who received prior erlotinib | Erlotinib 150mg qd alone  Cabozantinib 60mg qd alone  Erlotinib 150mgqd and Cabozantinib 60mg qd  Erlotinib 150mg qd and Cabozantinib 40mg qd  Cabozantinib 100mg qd  Cabozantinib 100mg qd alone  Cabozantinib 100mg qd and Erlotinib 50mg qd | 42  40  43  37  60  15  13 | 3%  11%  3%  10.8%  10%  6.7%  0% | 1.8 months  4.3 months  4.7 months  3.6 months  4.2 months  1.9 months  3.9 months |
| Foretinib | NCT01068587 | Advanced NSCLC | Foretinib 30-45mg qd and Erlotinib 100-150mg qd | 31 | 17.8% | Not reported |
| Tivantinib | NCT01395758  NCT01580735  NCT01377376, ATTENTION  NCT01244191, MARQUEE | KRAS-mutant advanced NSCLC  EGFR-mutant, metastatic or locally advanced NSCLC with progression on prior EGFR TKI  Locally advanced or metastatic, wild-type EGFR NSCLC  Previously treated, locally advanced or metastatic NSCLC | Tivantinib 360mg BID and Erlotinib 150mg qd  Single-agent chemotherapy, investigator’s choice  Tivantinib 240mg or 360mg BID and Erlotinib 150mg QID  Tivantinib 240mg or 360mg BID and Erlotinib 150mg qd  Erlotinib 150mg qd and placebo  Tivantinib 360mg BID and Erlotinib 150mg qd  Erlotinib 150mg qd and placebo | 51  45  45  154  153  526  522 | 0%  4.4%  6.7%  8.4%  6.5%  10.3%  6.5% | 1.7 months  4.3 months  2.7 months  2.9 months  2.0 months  3.6 months  1.9 months |

Supplemental Table 2. Active Clinical Trials of Type II and Type III MET TKIs

| Drug | Trial | Phase | Patient population | Treatment arms |
| --- | --- | --- | --- | --- |
| Cabozantinib | NCT03911193, CABinMET  NCT01639508  NCT03468985  NCT04471428, CONTACT-01 | II  II  II  III | NSCLC with MET amplification or MET exon 14 skipping mutation  Advanced NSCLC with RET or NTRK fusion or MET or AXL overexpression, amplification, or mutation  Recurrent stage IV NSCLC  Metastatic NSCLC previously treated with anti-PD-L1/PD-1 and platinum-containing chemotherapy | Cabozantinib PO qd  Cabozantinib PO qd  Nivolumab IV q28d alone  Nivolumab IV q28d and Cabozantinib PO qd  Nivolumab IV q28d and Cabozantinib PO qd and Ipilimumab IV q8w  Cabozantinib PO qd and Atezolizumab IV q28d  Docetaxel IV q21d |
| Merestinib | NCT02920996 | II | NSCLC with MET exon 14 skipping mutation | Merestinib PO qd |
| Glesatinib | NCT02954991  NCT02544633 | II  II | Advanced or metastatic NSCLC  Locally advanced, unresectable, or metastatic NSCLC with MET amplification or activating mutation | Glesatinib PO BID and Nivolumab IV q2w  Glesatinib PO BID |
| Tivantinib | NCT01069757  NCT01251796  NCT02049060 | I  I  I | Advanced/recurrent NSCLC  Advanced/recurrent NSCLC in CYP2C19 poor metabolizers  Advanced or metastatic NSCLC or malignant pleural mesothelioma | Tivantinib PO BID and Erlotinib PO qd  Tivantinib PO BID and Erlotinib PO qd  Tivantinib PO BID and Carboplatin IV q3w and Pemetrexed IV q3w |
